# Systems Approach to Study Associations between OxLDL and Abdominal Aortic Aneurysms

**DOI:** 10.3390/ijms20163909

**Published:** 2019-08-11

**Authors:** Łukasz Gutowski, Kaja Gutowska, Maria Pioruńska-Stolzmann, Piotr Formanowicz, Dorota Formanowicz

**Affiliations:** 1 Department of Clinical Biochemistry and Laboratory Medicine, Poznan University of Medical Sciences, Rokietnicka 8, 60-806 Poznan, Poland; 2 Institute of Computing Science, Poznan University of Technology, Piotrowo 2, 60-965 Poznan, Poland; 3 Institute of Bioorganic Chemistry, Polish Academy of Sciences, Noskowskiego 12/14, 61-704 Poznan, Poland

**Keywords:** abdominal aortic aneurysm, atherosclerosis, ldl, oxldl, modeling, petri nets, *t*-invariants

## Abstract

Although abdominal aortic aneurysm (AAA) is a common vascular disease and is associated with high mortality, the full pathogenesis of AAA remains unknown to researchers. Abdominal aortic aneurysms and atherosclerosis are strongly related. Currently, it is more often suggested that development of AAA is not a result of atherosclerosis, however, individual factors can act independently or synergistically with atherosclerosis. One of such factors is low-density lipoprotein (LDL) and its oxidized form (oxLDL). It is known that oxLDL plays an important role in the pathogenesis of atherosclerosis, thus, we decided to examine oxLDL impact on the development of AAA by creating two models using Petri-nets. The first, full model, contains subprocess of LDL oxidation and all subprocesses in which it participates, while the second, reduced model, does not contain them. The analysis of such models can be based on *t*-invariants. They correspond to subprocesses which do not change the state of the modeled system. Moreover, the knockout analysis has been used to estimate how crucial a selected transition (representing elementary subprocess) is, based on the number of excluded subprocesses as a result of its knockout. The results of the analysis of our models show that oxLDL affects 55.84% of subprocesses related to AAA development, but the analysis of the nets based on knockouts and simulation has shown that the influence of oxLDL on enlargement and rupture of AAA is negligible.

## 1. Introduction

An abdominal aortic aneurysm can occur in 5% of men aged 65–74 [1] and is associated with over 80% mortality rate after its rupture [2]. In 2013 rupture of the aortic aneurysm caused more than 150,000 deaths, making this disease the thirteenth leading cause of death in the world [1]. An abdominal aortic aneurysm is defined as an enlargement exceeding 1.5 times the normal size in a given segment [3]. The full mechanism of pathogenesis of the abdominal aortic aneurysm is still unknown.

Atherosclerosis and abdominal aortic aneurysms are strongly interrelated, and therefore, they cause controversy as to the individuality of these diseases [4,5]. Currently, it is more often suggested that the development of abdominal aortic aneurysm is not a result of atherosclerosis, and there are also separate factors that can act independently or synergistically with atherosclerosis [2]. Both disorders, on the other hand, share some risk factors and some similar pathological characteristics [5,6]. There are, therefore, questions about the factors that can be shared in the development of both disorders. One of such factors is LDL and its oxidized form—oxLDL. It is known that oxLDL plays an important role in the pathogenesis of atherosclerosis [7]. Nonetheless, studies about its role in AAA are inconclusive. Some of these studies do not indicate a relationship [2,8,9], while others report LDL participation in the development of abdominal aortic aneurysms [10,11,12]. According to our best knowledge there is no animal model describing influence of oxLDL on AAA, but the impact of oxLDL on the development of other aneurysms has been proven [13]. Therefore, there is a possibility of synergistic development of both diseases, which we decided to examine by creating a model of oxLDL influence on the development of AAA using the Petri-nets.

It is not always easy to study certain aspects of aneurysm on human tissues, that is why animal AAA models are used. There are several types of animal AAA models, however three of them are mainly used: elastase perfusion, CaCl${}_{2}$ application, and infusion of angiotensin II into either ApoE${}^{-/-}$. or LDL receptor${}^{-/-}$ mice. In the elastase model abdominal aorta is perfused with this elastase for a short period of time. It leads to dilatation after about 14 days and development of a chronic inflammatory response in aortic wall. Another model uses CaCl${}_{2}$. Calcium chloride is applied to mice by the peri-aortic incubation. In this model mice develop aneurysm at the end of the third week, and it is characterized by a chronic inflammatory response. In the third model, subcutaneous infusion of AngII into either LDL receptor${}^{-/-}$ or ApoE${}^{-/-}$ mice leads to the formation of AAAs in the suprarenal region within the 28-day infusion period. This method is the most widely used because it does not require abdominal surgery. Depending on the studies mice were fed with normal or high fat diet [6,11,12].

The model proposed by us describes interdependencies between many processes, such as the formation of oxLDL, production of reactive oxygen species by nitric oxide synthase (NOS) and NADPH oxidase enzymes (NOX) and their effect on the development of inflammation, increase in the amount of the metalloproteinases, and as a result - development and rupture of AAA.

The most important reactive form of oxygen in aneurysm development is superoxide anion radical (O${}_{2}^{•-})$ [14,15]. It is synthesized by uncoupled inducible NOS (iNOS) and endothelial NOS (eNOS), and NOX [15]. NOS with cofactor tetrahydrobiopterin (BH${}_{4}$)produces nitric oxide (NO) in physiological conditions. However, the oxidation of BH${}_{4}$ to BH${}_{2}$ under oxidative stress conditions leads to uncoupling of the enzyme and production of O${}_{2}^{•-}$. This radical intensifies the uncoupling of the enzyme, although it comes to it much faster under the influence of peroxynitrite (ONOO${}^{-}$), which is produced as a result of the reaction of O${}_{2}^{•-}$ and NO [16,17]. OxLDL also affects NOS, leading to its dephosphorylation, [18,19,20], and consequently to the production of superoxide anion radical instead of NO. Superoxide anion radical is reduced to hydrogen peroxide (H${}_{2}$O${}_{2}$) by enzyme dismutase (SOD) [21]. H${}_{2}$O${}_{2}$ is decomposed to water and oxygen by the enzyme catalase. O${}_{2}^{•-}$, H${}_{2}$O${}_{2}$ and ONOO${}^{-}$ are reactive oxygen and nitrogen species (ROS/RNS) that have a significant impact on the development of oxidative stress, which plays a significant role in the pathogenesis of AAA. Polymorphonuclear leukocytes (PMNs) release myeloperoxidase (MPO) which produces another ROS—hypochlorous acid (HOCl) from H${}_{2}$O${}_{2}$ and Cl${}^{-}$. HOCl indirectly stimulates proteolytic activity. MPO products can lead to lipid peroxidation [22]. ROS regulate Akt kinase activity involved in the process of vascular smooth muscle cells (VSMC) apoptosis [23,24,25], and activate activator protein-1 (AP-1) through MAPK, which leads to the production of chemokines and proinflammatory cytokines [24,26]. The most important cytokines in this disease entity are IL-6, IL-1β, IL-8 and TNFα. Production of some of them, like IL-6, could be increased, amongst others, by aortic wall stretch [27]. ROS damage DNA, which leads to in nuclear factor κB (NFκB) activity. NFκB also leads to the production of pro-inflammatory chemokines and cytokines as well as adhesion molecules: intercellular adhesion molecule-1 (ICAM-1) and vascular cell adhesion molecule-1 (VCAM-1) [24]. Oxidative stress increases the expression of an angiotensin-converting enzyme (ACE) [23]. It participates in the conversion process of angiotensin I in II. Angiotensin II activates NFκB and NOX by activating its subunits [24]. NOX is also stimulated by tumor necrosis factor α (TNFα), oxLDL and hemodynamic stress [28,29]. TNFα also activates NFκB [30]. ROS can oxidize LDL to oxLDL. OxLDL activates superoxide anion generation by NOX by activating its subunits [7,31]. OxLDL also affects on increase of O${}_{2}^{•-}$ concentration in other ways: it leads to dephosphorylation of NOS (as a result of which O${}_{2}^{•-}$ is produced, instead of NO) [18,19,20], and inhibits dismutase activity (decreases O${}_{2}^{•-}$ reduction to H${}_{2}$O${}_{2}$) [7]. OxLDL increases activity of metalloproteinase 2 (MMP2) [31] and the production of adhesion particles ICAM and VCAM [20].

Increase in adhesive particles and chemokines amount foster infiltration of the aortic wall by inflammatory cells. These cells intensify the synthesis of proinflammatory cytokines, which deepens inflammation and leads to the synthesis of proteases, mostly metalloproteinases [32,33,34]. Reactive oxygen species also take part in the expression of genes and activation of latent MMPs [10]. The essential metalloproteinases in the pathogenesis of AAA are 1, 2, 7, 9, 12, 13. They degrade connective tissues. The proteolysis products are chemoattractants, intensifying infiltration by inflammatory cells [35]. MMPs 2, 7, 9, and 12 are mainly responsible for the degradation of elastin which results in aortic dilatation [35]. This process is intensified by previously mentioned VSMC apoptosis because these cells are responsible for the synthesis of the elastin [17,35]. Enlargement of the aneurysm increases the hemodynamic stress, which leads to compensatory deposition of collagen in the aortic wall. This makes it possible to maintain resistance to the pressure exerted on the vessel wall [35]. In turn, MMPs 1, 8, and 13 are mainly responsible for the degradation of collagen. If collagen predominate in the aortic wall, the long-term effects of metalloproteinases 1, 8 and 13 may result in rupture of the aneurysm.

The description above shows very complex biological phenomenon, which should also be treated as a complex biological system. To understand this phenomenon better, systematize knowledge, discover new properties, and confirm specific facts, the systems approach has been used. In this study, a systems approach based on Petri-net, and its analysis based on *t*-invariants has been applied.

## 2. Analysis

The presented analysis of the proposed Petri-net models is based on *t*-invariants. Two main parts of such an analysis can be distinguished, i.e., an analysis of Maximum Common Transition sets (MCT sets) and an analysis of *t*-clusters. In addition to classical analysis, significance and knockout analyses were performed. The former one determines percentage of all subprocesses in which selected elementary subprocesses are involved. Such an analysis allows to distinguish which subprocesses are more crucial for functioning of the modeled system. As a complement, the knockout analysis allows to estimate how crucial selected transition is, based on the number of excluded subprocesses. The knockout simulation collects more detailed data about a behavior of the net when selected transitions are excluded.

The analysis of MCT sets is associated with determination of certain functional blocks. For the presented full model, 15 MCT sets were described in Table A3 in Appendix B. A need of the analysis of *t*-clusters follows from a large number of *t*-invariants. They were grouped in such clusters and a biological meaning of each of them was determined. For the proposed Petri-net 17 *t*-clusters described in Table A4 in Appendix C were calculated. In this case, analysis of *t*-clusters does not allow for accurate analysis of all subprocesses. Therefore, a significance analysis was performed for selected transitions (elementary subprocess). This means that for each transition, its attendance frequency is determined in all supports of *t*-invariants.

To complement the analysis of selected *t*-invariants a knockout analysis was performed (cf. [36,37]). This analysis relies on disabling selected transition (elementary subprocess/selected reaction). Turning off certain active component of a Petri-net consequently leads to exclusion of others. Knockouted transition (certain reaction) may disable large fragments of the net, when it plays a key role in the functioning of the entire model. After turning off the selected transition or several transitions, *t*-invariants are calculated again. On this basis it is possible to estimate which subprocesses have been excluded in consequence of knockout of selected transitions. Thus, the knockout analysis allows to estimate how crucial selected transition (elementary subprocess) is, based on the number of excluded subprocesses.

These analyses focuses on subprocesses having an indirect and direct impact on the development and rupture of the abdominal aortic aneurysm. Distinguished subprocesses are: LDL oxidation, oxidative stress, production of inflammatory cytokines, influence of MMPs, and impact of NOX. The results were presented for both full model and reduced model, to assess the impact of oxLDL on the development of AAA on this basis. The results of the significance analysis of selected subprocesses for full model and reduced model are presented in Table 1. Selected subprocesses may often consist of more than one elementary subprocess (transition). On this basis, it is possible to determine the percentage of all subprocesses in which a specific elementary subprocess is involved. From a mathematical point of view it is determined in how many *t*-invariants a selected transition occurs. In consequence, conclusions which subprocesses are more crucial for functioning of the modeled system than others can be drawn. In Table 1, it can be seen that subprocess of oxLDL appears in 55.84% of all modeled subprocesses in full model. This result could suggest that oxLDL has high significance for whole system. Nonetheless, knockout analysis was also conducted to achieve full analysis.

Table 1 includes information about percentage contribution of particular elementary subprocess (single transition) in the whole system, while Table 2 contains percentage contribution of particular subprocess. To be precise, Table 2 contains results of knockout analysis of selected subprocesses for two models: full and reduced. As mentioned before, some subprocesses consist of several elementary subprocesses (several transitions), and all of them are turned off in this analysis. Knockout of such set of transitions allows to determine contribution of certain subprocess in the whole system. Excluded sets of transitions are indicated in the row called “knockouted transitions”. It should be noted that the reduced model does not include certain transitions, inter alia, $t_{0}$ which is associated with LDL oxidation, hence no result in this field of Table 2. Thus, comparing full model (with oxLDL) and reduced model (without oxLDL) allows to estimate the percentage contribution of oxLDL in the selected subprocesses, i.e., oxidative stress, influence of MMPs, influence of NOX, production of inflammatory cytokines, enlargement and rupture of AAA.

Table 3 summarizes the most important results of the analysis of the proposed full model. For subprocesses associated with enlargement and rupture of aneurysm, the significance of a given subprocess in the full network (736 *t*-invariants) and in the knockouted network (325 *t*-invariants) is presented. In the last column, the difference between percentage contribution in the whole system for full model, and full model with knockout was calculated. On this basis influence of oxLDL on aneurysm enlargement (1.15 p.p.) and rupture (2.71 p.p.) has been determined. It can be noticed that the knockout analysis for the full model (excluded transition $t_{0}$ is associated with LDL oxidation) gives the same results as the significance analysis (Table 1) for the reduced Petri-net model. As can be seen, results suggest that these analyzes are consistent.

To complete the above analyses and confirm the results that the influence of oxLDL on aneurysm enlargement is only 1.15 p.p. and aneurysm rupture is 2.71 p.p., a knockout simulation was performed. Its purpose was collection of more detailed data about behavior of Petri-net in situation of excluding various transitions and comparing them with each other. This simulation can be performed in the Java application called Holmes [38].

The simulation was carried out for full model and for full model with knockout (knockouted transition $t_{0}$—LDL oxidation). Simulation properties are as follows: 10,000 steps, 1000 repetitions. On this basis, we obtained average number of firing of transition (AvgF) in all steps in all simulations for these two models. Our results focus on transitions $t_{52}$ (enlargement of AAA) and $t_{54}$ (rupture of AAA):

- AvgF for $t_{52}$ in full model is 19.40, while AvgF for $t_{52}$ in full model with knockout of $t_{0}$ is 19.46.
- AvgF for $t_{54}$ in full model is 4.85, while AvgF for $t_{54}$ in full model with knockout of $t_{0}$ is 4.86.

As it can be noticed, differences in the average number of firings of transitions $t_{52}$ and $t_{54}$ before and after knockout are insignificant. This confirms the observations summarized in Table 3. All key subprocesses have not changed after the knockout, which means that the subprocesses of enlargement and rupture of AAA can occur without influence of oxLDL.

## 3. Petri-Net-Based Models

**Figure 1:**
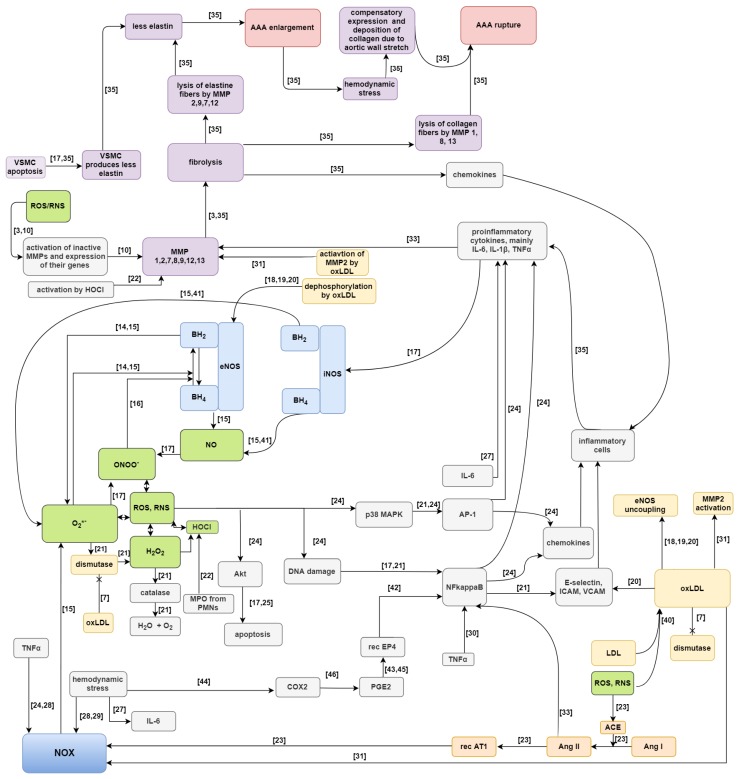
Scheme of the proposed full model of AAA development. References was marked above the arrows for analysis facilitation [3,7,10,14,15,16,17,18,19,20,21,22,23,24,25,27,28,29,30,31,33,35,40,41,42,43,44,45,46].

Two Petri-net-based models of aneurysm formation and rupture have been proposed (they are extended versions of the net presented in [39]). These models have been created using Holmes. The first, full model, contains subprocess of LDL oxidation to oxLDL and all subprocesses in which they participate, i.e., stimulation of NOX, stimulation of adhesion particles, inhibition of SOD, activation of elastin proteolysis by MMP2, and synthesis of superoxide anion radical together with NOS. While the second, reduced model, does not contain these subprocesses. These two models of Petri-nets are compared to each other. The main purpose of such comparison is evaluation how large impact on the enlargement and rupture of AAA have subprocesses related to oxLDL. A Petri-net model, despite its intuitive graphical representation, is complicated due to a large number of passive and active components. Therefore, a schematic diagram is shown in Figure 1 [3,7,10,14,15,16,17,18,19,20,21,22,23,24,25,27,28,29,30,31,33,35,40,41,42,43,44,45,46]. In this scheme of AAA development green color indicates reactive oxygen/nitrogen species, NOX and NOSs are marked blue, participation of angiotensin is marked orange, processes associated with oxLDL are marked yellow, factors affecting enlargement and rupture of AAA are marked purple, and AAA enlargement and rupture are marked with pink color.

**Figure A1:**
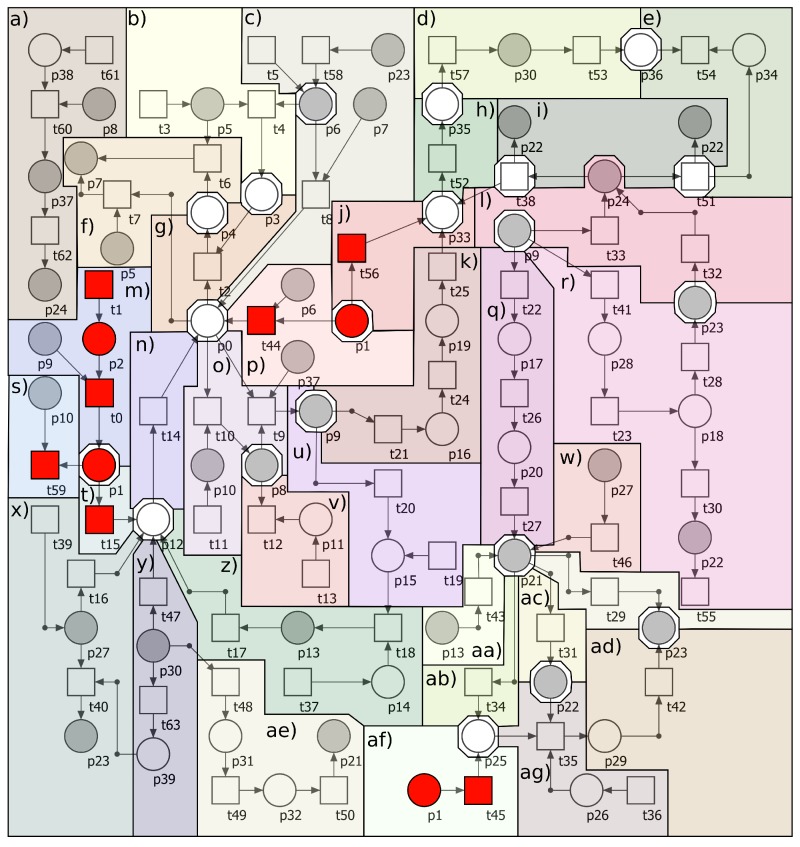
The Petri-net-based model of formation and rupture of abdominal aortic aneurysm including subprocesses associated with oxLDL (full model, with oxLDL). The model was divided into several blocks: (a) production of HOCl, (b) synthesis of NO, (c) synthesis of O${}_{2}^{•-}$ by NOSs and BH${}_{2}$, (d) compensating collagen deposition by hemodynamic stress, (e) rupture of AAA, (f) oxidation of BH${}_{4}$ to BH${}_{2}$, (g) synthesis of ONOO${}^{-}$ by O${}_{2}^{•-}$ and NO, (h) enlargement of AAA, (i) proteolysis of elastin and collagen by MMPs, (j) additional activation of MMP2 by oxLDL, (k) VSMC apoptosis, (l) expression of MMPs by inflammatory cytokines and ROS, (m) oxidation of LDL, (n) synthesis of O${}_{2}^{•-}$ by NOX, (o) dismutation of O${}_{2}^{•-}$ into H${}_{2}$O${}_{2}$, (p) synthesis of O${}_{2}^{•-}$ by NOSs and oxLDL, (q) activation of NFκB caused by DNA damage by ROS, (r) stimulation of AP1 by ROS, (s) inhibition of SOD by oxLDL, (t) stimulation of NOX by oxLDL, (u) activation of ACE by ROS, (v) reduction of H${}_{2}$O${}_{2}$ by catalase, (w) stimulation of NFκB by TNFα, (x) activation of NOX by TNFα, (y) activation of NOX, COX2 and IL-6 by hemodynamic stress, (z) activation of NOX by ANGII (conversion of ANGI to ANGII by ACE), (aa) stimulation of NFκB by ANGII, (ab) production of adhesion particles by NFκB, (ac) stimulation of chemokines production by NFκB, (ad) stimulation of inflammatory cytokines production by NFκB, (ae) activation of NFκB via PGE2 activated by COX2, (af) stimulation of adhesion particles by oxLDL, (ag) diapedesis, (ah) infiltration by inflammatory cells. In addition, transitions and places related to LDL oxidation and their roles in the modeled process were marked with red squares and circles. These specific elements were omitted in the second model (reduced model, without oxLDL). In addition, some places and transitions that are common for the highlighted blocks are marked with a non-colored octagon.

The proposed full model of Petri-net is shown in Figure A1 in Appendix A. In that figure oxLDL subprocesses, marked with red color, are included in the full model, and they were removed from reduced model.

Petri-net-based full model of AAA development contains 64 transitions and 40 places. Places correspond to passive components of the modeled system, while transitions correspond to its active components. Descriptions of all places and transitions of the net are included in Appendix A in Table A1 and Table A2, respectively. Full model is covered by 736 *t*-invariants (which correspond to biological subprocesses. Reduced model, which does not contains subprocesses related to oxLDL, contains 57 transitions and 38 places. Transitions and places that have been removed from this model are marked with red in Figure A1 and their names are marked in bold font in Table A1 and Table A2 in Appendix A. Reduced model is covered by 325 *t*-invariants.

## 4. Methods

Petri-nets are mathematical objects suitable for modeling a wide class of systems, especially those ones which contain concurrent processes. They were proposed in 1962 by Carl A. Petri in the context of computer science [47]. Such nets have been used for years in modeling and analysis of technical systems. In the mid of 1990s it has been realised that nets of this type can be used also for investigations of properties of biological systems (cf. [48]).

Petri-nets have a structure of a directed bipartite graph what means that they are composed of two disjoint subsets of vertices, called places and transitions. These vertices are connected by arcs in such a way that an arc connects a place with a transition or a transition with a place (i.e., no two places nor two transitions are connected). When a Petri-net is a model of a biological system places correspond to its passive components, as chemical compounds, while transitions are counterparts of active components, as chemical reactions. Arcs describe causal relations between the passive and active components and they are labeled by positive integer numbers called weights [49,50].

There is one more type of components of Petri-nets, i.e., tokens. They bring into the net dynamics (which is crucial for modeling systems) not only the biological ones. Tokens flow from one place to another through transitions. This flow corresponds to a flow of substances, information etc. through the modeled system. It is governed by a simple rule of transition firing. According to this rule transition $t_{j}$ is active if in every place $p_{i}$ directly preceding it (such a place is called a pre-place of transition $t_{j}$) the number of tokens is equal to at least the weight of arc $\left(p_{i},t_{j}\right)$, i.e., the arc connecting $p_{i}$ with $t_{j}$. An active transition can be fired, what means that tokens flow from its pre-places to its post-places, i.e, the places directly succeeding $t_{j}$, and the numbers of flowing tokens are equal to weights of the appropriate arcs [49,50].

Petri-nets have an intuitive graphical representation. In this representation places are depicted as circles, transitions as rectangles, arcs as arrows and tokens as dots or numbers residing in places. When a weight of an arc is equal to one, it is not shown in the graphical representation of the net. While this representation is very helpful in understanding the structure of the modeled system and supports simulations of its behavior, it is not very well suited for a formal analysis of its properties. For this purpose another representation, called an incidence matrix, can be used. In such a matrix $A={\left(a_{ij}\right)}_{n\times m}$, where *n* is a number of places and *m* is a number of transitions, entry $a_{ij}$ is equal to a difference between numbers of tokens in place $p_{i}$ before and after firing transition $t_{j}$ [48].

In the analysis of Petri-net-based models of biological systems especially important are *t*-invariants, which are vectors $x\in Z^{m}$ being solutions to equation A·x=0. To every *t*-invariant there corresponds set of transitions $s\left(x\right)=\left\{t_{j}:x_{j}>0\right\}$, called its support. When a Petri-nets is a model of a biological system, usually it should be covered by *t*-invariants, what means that each transition should be an element of a support of some such an invariant. *t*-invariants are counterparts of subprocesses which do not change a state of the modeled system. More precisely, if every transition $t_{j}\in s\left(x\right)$ is fired $x_{j}$ times, a distribution of tokens in places (called a marking of the net) does not change $t_{j}$ [48].

From this follows that an analysis of similarities among *t*-invariants may lead to discoveries of previously unknown properties of the modeled system. Indeed, such similarities may be a reason of interactions between the above mentioned subprocesses, since supports of similar *t*-invariants have a non-empty intersection. This intersection contains some transitions corresponding to elementary processes being components of the subprocesses. Since these subprocesses contain some common elementary processes they can interact with each other through them. Hence, looking for similar *t*-invariants may lead to identifying unknown interactions of subprocesses which may be a source of some important properties of the analyzed biological system [51,52,53].

Searching for similarities among *t*-invariants can be done using standard clustering algorithms. They divide the set of all *t*-invariants into a disjoint subset containing *t*-invariants which are similar to each other according to some similarity measure. However, it is not a trivial task since a proper algorithm, a proper similarity measure and a proper number of clusters should be chosen. All of these three components of the clustering procedure should be adjusted to the modeled system and to the problem to be solved on the basis of the model. The resulting clusters are called *t*-clusters and correspond to some functional modules of the biological system.

Moreover, also the set of transitions can be divided into disjoint subsets called MCT sets. Such a set contains transitions being elements of supports of the same *t*-invariants and corresponds to some functional block of the modeled biological system. During the analysis biological meaning of MCT sets as well as *t*-clusters should be determined (cf. [53,54]).

**Figure 2:**
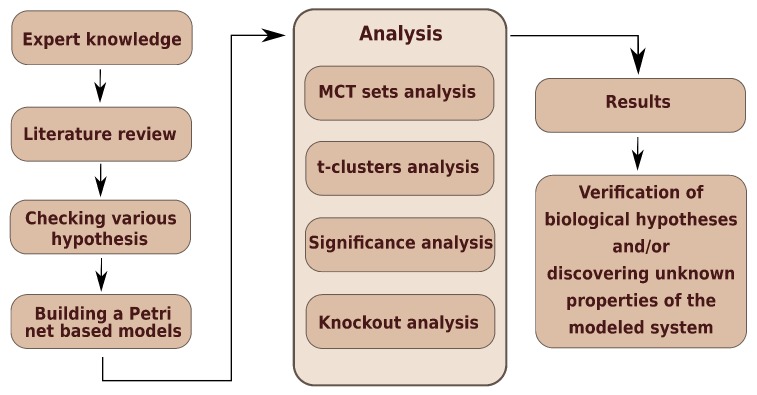
The general scheme of work.

Figure 2 presents a general scheme of work, where three stages can be identified. The first—creation of model, the second—analyses of model, and the third—result obtainment.

## 5. Conclusions

There is no agreement in the scientific community about the influence of LDL and its oxidized form on the formation of abdominal aortic aneurysms. According to popular belief, it might seem that oxLDL should be involved, not only in the pathogenesis of atherosclerosis, but also in the pathogenesis of abdominal aortic aneurysm, however knockout analysis and simulation of the proposed Petri-net-based models with and without oxLDL have shown that there is almost no influence of oxLDL on aneurysm enlargement and rupture. Nevertheless, it should be noted that in our model oxLDL is associated with 55.84% of all modeled subprocesses involved in the pathogenesis of aneurysm. Despite the involvement of oxLDL in more than half of the subprocesses, its participation does not significantly translate into enlargement or rupture of aneurysm. Our results seem to reconcile some of the contradictory reports: oxLDL is indeed involved in the formation of aneurysms, but it is not reflected significantly in its enlargement or rupture, and the whole process can take place without its participation.

## Figures and Tables

**Table 1:** Significance analysis of selected subprocesses for full and reduced model. The following abbreviations in the columns headings has been distinguished: trans—transition, inv— *t*-invariant, frequency trans/inv—frequency of occurrence of selected transition in all *t*-invariants.

		Full Model with oxLDL		Reduced Model without oxLDL	
Subprocess	Elementary Subprocess	Frequency Trans/Inv	Percentage of Transition in Whole System (100% = 736 inv)	Frequency Trans/Inv	Percentage of Transition in Whole System (100% = 325 inv)
	production of O${}_{2}^{•-}$ by NOX	713	96.88%	302	92.92%
	production of O${}_{2}^{•-}$ by NOS and oxLDL	205	27.85%	-	-
oxidative stress	production of O${}_{2}^{•-}$ by NOS and BH${}_{2}$	364	49.46%	218	67.08%
	production of ONOO${}^{-}$ by O${}_{2}^{•-}$ and NO	182	24.73%	109	33.54%
	production of H${}_{2}$O${}_{2}$ through dismutation	713	96.88%	302	92.92%
	production of HOCl by MPO and H${}_{2}$O${}_{2}$	707	96.06%	296	91.08%
	activation by ROS	197	26.77%	88	27.08%
	activation by cytokines	192	26.09%	99	30.46%
influence of MMPs	activation of MMP2 by oxLDL	132	17.93%	-	-
	activation of MPO by HOCl	184	25.00%	75	23.08%
LDL oxidation	LDL oxidation	411	55.84%	-	-
	activation by oxLDL	6	0.82%	-	-
influence of NOX	activation by TNFα	342	46.47%	143	44.00%
	activation by hemodynamic stress	145	19.70%	79	24.31%
	activation by ANGII	291	39.54%	115	35.38%
	production by inflammatory cells	552	75.00%	211	64.92%
	production by TNFα	108	14.67%	53	16.31%
production of inflammatory cytokines	production by NFκB	115	15.63%	55	16.92%
	production by AP1	26	3.53%	20	6.15%
	stimulation of IL-6	108	14.67%	53	16.31%
enlargement of AAA	enlargement of AAA	629	85.46%	274	84.31%
rupture of AAA	rupture of AAA	260	35.33%	106	32.62%

**Table 2:** Knockout analysis of selected subprocesses for full and reduced model.

	Oxidative Stress	Influence of MMPs	LDL Oxidation	Influence of NOX	Production of Inflammatory Cytokines	Enlargement of AAA	Rupture of AAA
full model (with oxLDL)	98.78%	80.84%	55.84%	96.88%	93.75%	85.46%	35.33%
reduced model (without oxLDL)	97.23%	78.15%	-	92.92%	90.15%	84.31%	32.62%
knockouted transitions	$t_{2}$, $t_{8}$, $t_{10}$, $t_{14}$, $t_{44}$, $t_{60}$	$t_{32}$, $t_{33}$, $t_{56}$, $t_{62}$	$t_{0}$	$t_{15}$, $t_{16}$, $t_{17}$, $t_{47}$	$t_{28}$, $t_{29}$, $t_{40}$, $t_{42}$, $t_{63}$	$t_{52}$	$t_{54}$

**Table 3:** The key results from analysis.

Full Model with oxLDL					
	Significance Analysis of Selected Subprocesses		Knockout of Full Model (Knockout Transition $t_{0}$)		
Subprocess	Frequency Trans/Inv	Percentage Contribution in Whole System (100% = 736 inv)	Frequency Trans/Inv	Percentage Contribution in Whole System (100% = 325 inv)	Difference between Percentage Contribution (736 inv vs. 325 inv)
enlargement of AAA	629	85.46%	274	84.31%	1.15 p.p.
rupture of AAA	260	35.33%	106	32.62%	2.71 p.p.

## Appendix A. The Proposed Petri Net Model

**Table A1:** List of places for full model.

Place	Biological Meaning	Place	Biological Meaning
$p_{0}$	superoxide anion radical	$p_{20}$	PKC
$p_{1}$	**oxidized LDL**	$p_{21}$	NFκB
**$p_{2}$**	**LDL**	$p_{22}$	chemokines
$p_{3}$	NO	$p_{23}$	inflammatory cytokines
$p_{4}$	peroxynitrite	$p_{24}$	MMPs
$p_{5}$	BH${}_{4}$	$p_{25}$	adhesion particles
$p_{6}$	NOSs	$p_{26}$	circulating inflammatory cells
$p_{7}$	BH${}_{2}$	$p_{27}$	TNFα
$p_{8}$	H${}_{2}$O${}_{2}$	$p_{28}$	p38MAPK
$p_{9}$	ROS	$p_{29}$	active inflammatory cells
$p_{10}$	dismutase	$p_{30}$	hemodynamic stress
$p_{11}$	catalase	$p_{31}$	COX2
$p_{12}$	NOX	$p_{32}$	PGE2
$p_{13}$	ANGII	$p_{33}$	less elastin
$p_{14}$	ANGI	$p_{34}$	less collagen
$p_{15}$	ACE	$p_{35}$	enlarged AAA
$p_{16}$	tyrosine kinase	$p_{36}$	deposited collagen
$p_{17}$	PARP polymerase	$p_{37}$	HOCl
$p_{18}$	AP1	$p_{38}$	MPO
$p_{19}$	Akt	$p_{39}$	IL-6

**Table A2:** List of transitions for full model.

Transition	Biological Meaning	Transition	Biological Meaning
**$t_{0}$**	**LDL oxidation**	$t_{32}$	expression of MMPs by inflammatory cytokines
**$t_{1}$**	**blood as LDL source**	$t_{33}$	activation of MMPs by ROS
$t_{2}$	peroxynitrite synthesis	$t_{34}$	production of adhesion particles
$t_{3}$	BH${}_{4}$ synthesis	$t_{35}$	diapedesis
$t_{4}$	NO synthesis	$t_{36}$	source of inflammatory cells
$t_{5}$	NOSs sources	$t_{37}$	source of ANGI
$t_{6}$	BH${}_{4}$ oxidation to BH${}_{2}$ through peroxynitrite	$t_{38}$	elastine proteolysis by MMPs
$t_{7}$	BH${}_{4}$ oxidation to BH${}_{2}$ through O${}_{2}^{•-}$	$t_{39}$	source of TNFα
$t_{8}$	O${}_{2}^{•-}$ synthesis via NOSs and BH${}_{2}$	$t_{40}$	pool of inflamatory cytokines
$t_{9}$	pool of ROS	$t_{41}$	activation of p38MAPK
$t_{10}$	dismutation	$t_{42}$	production of inflammatory cytokines by inflmmatory cells
$t_{11}$	source of dismutase	$t_{43}$	direct stimulation of NFκB via ANGII
$t_{12}$	H${}_{2}$O${}_{2}$ reduction	**$t_{44}$**	**O${}_{2}^{•-}$ synthesis via NOSs and oxLDL**
$t_{13}$	source of catalase	**$t_{45}$**	**stimulation of adhesion particles**
$t_{14}$	O${}_{2}^{•-}$ synthesis through NOX	$t_{46}$	stimulation of NFκB by TNFα
**$t_{15}$**	**indirect stimulation of NOX through oxLDL**	$t_{47}$	activation of NOX by hemodynamic stress
$t_{16}$	activation of NOX through TNFα	$t_{48}$	activation of COX2 by hemodynamic stress
$t_{17}$	activation of NOX by ANGII	$t_{49}$	production of PGE2 by COX2
$t_{18}$	conversion of ANGI to ANGII	$t_{50}$	activation of NFκB via PGE2
$t_{19}$	source of ACE	$t_{51}$	collagen proteolysis by MMPs
$t_{20}$	activation of ACE by ROS	$t_{52}$	enlargement of AAA
$t_{21}$	stimulation of tyrosine kinase	$t_{53}$	compensating collagen deposition
$t_{22}$	DNA damage and release of PARP	$t_{54}$	rupture of AAA
$t_{23}$	stimulation of AP1	$t_{55}$	degradation of chemokines
$t_{24}$	indirect stimulation of Akt	**$t_{56}$**	**additional activation of elastin proteolysis by MMP2**
$t_{25}$	VSMC apoptosis	$t_{57}$	increase of hemodynamic stress
$t_{26}$	stimulation of PKC by PARP	$t_{58}$	activation of iNOS by inflammatory cytokines
$t_{27}$	activation of NFκB by PKC	**$t_{59}$**	**inhibition of SOD**
$t_{28}$	stimulation of inflammatory cytokines production by AP1	$t_{60}$	production of HOCl by MPO
$t_{29}$	stimulation of inflammatory cytokines production by NFκB	$t_{61}$	source of MPO
$t_{30}$	stimulation of chemokines production by AP1	$t_{62}$	activation of MMPs by HOCl
$t_{31}$	stimulation of chemokines production by NFκB	$t_{63}$	stimulation of IL-6

## Appendix B. Analysis of MCT Sets

Analysis of models based on Petri-net focuses mainly on *t*-invariants. On the basis of *t*-invariants, MCT sets are calculated. These sets divide Petri-net into blocks containing certain transitions. To be precise, these transitions are elements of supports of exactly the same *t*-invariants. From the biological point of view, MCT sets divide Petri-net into some functional blocks. Biological descriptions for all MCT sets are included in Table A3.

**Table A3:** Biological meaning of non-trivial MCT sets for full model.

MCT-Set	Contained Transitions	Biological Meaning
$m_{1}$	$t_{2}$, $t_{4}$, $t_{6}$	Oxidation of BH${}_{4}$ to BH${}_{2}$ by ONOO${}^{-}$.
$m_{2}$	$t_{10}$, $t_{11}$, $t_{14}$	Dismutation of NOX-derived O${}_{2}^{•-}$.
$m_{3}$	$t_{21}$, $t_{24}$, $t_{25}$	Apoptosis of VSMC.
$m_{4}$	$t_{22}$, $t_{26}$, $t_{27}$	Activation of NFκB caused by DNA damage by ROS.
$m_{5}$	$t_{35}$, $t_{36}$, $t_{42}$	Infiltration by inflammatory cells.
$m_{6}$	$t_{48}$, $t_{49}$, $t_{50}$	Activation of NFκB via PGE2 activated by COX2.
$m_{7}$	$t_{51}$, $t_{53}$, $t_{54}$	Collagen proteolysis and rupture of aneurysm.
$m_{8}$	$t_{0}$, $t_{1}$	Oxidation of LDL.
$m_{9}$	$t_{3}$, $t_{8}$	Synthesis of O${}_{2}^{•-}$ by NOS and BH${}_{2}$.
$m_{10}$	$t_{12}$, $t_{13}$	Reduction of H${}_{2}$O${}_{2}$ by catalase.
$m_{11}$	$t_{18}$, $t_{37}$	Conversion of ANGI to ANGII by ACE.
$m_{12}$	$t_{23}$, $t_{41}$	Stimulation of AP1 by ROS.
$m_{13}$	$t_{40}$, $t_{63}$	Stimulation of IL-6 by hemodynamic stress.
$m_{14}$	$t_{52}$, $t_{57}$	Influence of aneurysm enlargement on hemodynamic stress.
$m_{15}$	$t_{60}$, $t_{61}$	Production of HOCl by MPO and H${}_{2}$O${}_{2}$.

## Appendix C. Analysis of *t*-Clusters

As it has been already mentioned, analysis of Petri-nets can be based on *t*-invariants. Such invariants are crucial from biological point of view because they correspond to modeled subprocesses. In the case of a large number of *t*-invariants, the biological significance is not assigned to each of them. The proposed full model contains 736 *t*-invariants, therefore clustering was used. Biological significance was assigned for groups of *t*-invariants similar to each other (called *t*-clusters) and their descriptions are given in Table A4. 17 *t*-clusters for the proposed Petri-net were distinguished.

Clustering of *t*-invariants was performed by using a Java application called Holmes [38]. For clustering 7 different algorithms and 8 different distance measures has been used. The results presented in this paper have been obtained using a clustering algorithm based on the average linkage method and Pearson’s measure of distance. The clustering method, the distance measure and the number of *t*-clusters were selected on the basis of the MSS index (Mean Split Silhouette) and the C-H coefficient (Calinski-Harabasz) [55,56].

**Table A4:** Biological meaning of *t*-clusters for full model.

*t*-Cluster	Biological Meaning
$c_{1}$	Uncoupling of NOS by ONOO${}^{-}$ and O${}_{2}^{•-}$.
$c_{2}$	Influence of hemodynamic stress on activation of NFκB, which activates production of chemokines and inflammatory cytokines, and results in inflammatory cells infiltration. Inflammatory state leads to production of MMPs and enlargement of aneurysm.
$c_{3}$	This cluster includes all modeled subprocesses.
$c_{4}$	This cluster includes almost all modeled subprocesses except: activation of NOX through TNFα.
$c_{5}$	Activation of NFκB by TNFα. NFκB produces chemokines.
$c_{6}$	Influence of ANGII on enlargement and rupture of aneurysm.
$c_{7}$	Synthesis of HOCL by MPO leads to activation of MMPs which results in enlargement and rupture of aneurysm. Activation of NOX by ANGII, TNFα, hemodynamic stress. PGE2 stimulates NFκB which activates production of chemokines.
$c_{8}$	Activation of inflammatory cytokines (TNFα and IL-6) resulting in enlargement of aneurysm.
$c_{9}$	This cluster includes almost all modeled subprocesses except: Activation of NFκB via PGE2 activated by COX2. Activation of NOX through TNFα.
$c_{10}$	Activation of NFκB by TNFα. NFκB produces cytokines, which activates iNOS. iNOS and eNOS are uncoupled by ONOO${}^{-}$ and O${}_{2}^{•-}$.
$c_{11}$	Activation of NFκB by ANGII. NFκB activates production of chemokines and inflammatory cytokines, and results in inflammatory cells infiltration. Cytokines activates iNOS, iNOS and eNOS are uncoupled by ONOO${}^{-}$ and O${}_{2}^{•-}$.
$c_{12}$	Activation of NOX by ANGII and TNFα. PGE2 stimulates NFκB, which enhances tissue infiltration by inflammatory cells. Inflammatory cells produce cytokines resulting in activation of iNOS. Part of O${}_{2}^{•-}$ undergoes dismutation to H${}_{2}$O${}_{2}$, and other part reacts with NO leading to ONOO${}^{-}$ synthesis. ONOO${}^{-}$ leads to uncoupling of NOS. Synthesis of HOCL by MPO leads to activation of MMPs which results in enlargement of aneurysm.
$c_{13}$	Activation of NOX by ANGII, TNFα and hemodynamic stress. ANGII and TNFα stimulates NFκB, which enhances tissue infiltration by inflammatory cells. Inflammatory cells produce cytokines (mainly IL-6, IL-1 and TNFα) resulting in activation of iNOS. Part of O${}_{2}^{•-}$ undergoes dismutation to H${}_{2}$O${}_{2}$, and other part reacts with NO leading to ONOO${}^{-}$ synthesis. ONOO${}^{-}$ leads to uncoupling of NOS. Synthesis of HOCl by MPO leads to activation of MMPs which results in enlargement and rupture of aneurysm.
$c_{14}$	Activation of NFκB by TNFα. NFκB activates production of chemokines and adhesion particles, which results in inflammatory cells infiltration. Cytokines activates iNOS, iNOS and eNOS are uncoupled by ONOO${}^{-}$.
$c_{15}$	Activation of NFκB by ANGII and TNFα. TNFα and IL-6 as inflammatory cytokines lead to expression of MMPs and enlargement of aneurysm. Adhesion particles produced by NFκB and chemokines result in inflammatory cells infiltration. Cytokines activates iNOS, iNOS and eNOS are uncoupled by ONOO${}^{-}$.
$c_{16}$	Activation of NFκB by TNFα leads to inflammatory cytokines production. Inflammatory cytokines activates expression of MMPs, which results in enlargement and rupture of aneurysm.
$c_{17}$	This cluster includes almost all modeled subprocesses except: Activation of NFκB via PGE2 activated by COX2. Stimulation of IL-6 by hemodynamic stress. Activation of NOX through TNFα. Uncoupling of NOS by ONOO${}^{-}$ and O${}_{2}^{•-}$. Activation of MMPs by HOCl. Stimulation of chemokines production by NFκB. Production of adhesion particles. Rupture of aneurysm.
